# Progression of bone metastases in patients with prostate cancer - automated detection of new lesions and calculation of bone scan index

**DOI:** 10.1186/2191-219X-3-64

**Published:** 2013-08-16

**Authors:** Reza Kaboteh, Peter Gjertsson, Håkan Leek, Milan Lomsky, Mattias Ohlsson, Karl Sjöstrand, Lars Edenbrandt

**Affiliations:** 1 Department of Molecular and Clinical Medicine, Sahlgrenska Academy at the University of Gothenburg, Gothenburg SE 413 45, Sweden; 2 Department of Clinical Sciences, Lund University, Malmö, Sweden; 3 Department of Theoretical Physics, Lund University, Lund, Sweden; 4 EXINI Diagnostics AB, Lund, Sweden

**Keywords:** Image analysis, Radionuclide imaging, Bone metastases, Prostate cancer, Automated detection, Computer-assisted diagnosis

## Abstract

**Background:**

The objective of this study was firstly to develop and evaluate an automated method for the detection of new lesions and changes in bone scan index (BSI) in serial bone scans and secondly to evaluate the prognostic value of the method in a group of patients receiving chemotherapy.

**Methods:**

The automated method for detection of new lesions was evaluated in a group of 266 patients using the classifications by three experienced bone scan readers as a gold standard. The prognostic value of the method was assessed in a group of 31 metastatic hormone-refractory prostate cancer patients who were receiving docetaxel. Cox proportional hazards were used to investigate the association between percentage change in BSI, number of new lesions and overall survival. Kaplan-Meier estimates of the survival function were used to indicate a significant difference between patients with an increase/decrease in BSI or those with two or more new lesions or less than two new lesions.

**Results:**

The automated method detected progression defined as two or more new lesions with a sensitivity of 93% and a specificity of 87%. In the treatment group, both BSI changes and the number of new metastases were significantly associated with survival. Two-year survival for patients with increasing and decreasing BSI from baseline to follow-up scans were 18% and 57% (*p* = 0.03), respectively. Two-year survival for patients fulfilling and not fulfilling the criterion of two or more new lesions was 35% and 38% (n.s.), respectively.

**Conclusions:**

An automated method can be used to calculate the number of new lesions and changes in BSI in serial bone scans. These imaging biomarkers contained prognostic information in a small group of patients with prostate cancer receiving chemotherapy.

## Background

In the near future, there may be several treatment options for patients with castrate-resistant prostate cancer. Zoledronic acid [1] and docetaxel [2] were introduced 8 to 10 years ago, and for many years, no new drugs have been presented for this patient group. Recently, however, new agents such as sipuleucel-T [3], cabazitaxel [4], denosumab [5], abiraterone [6], and MDV3100 [7] have been proposed as new treatment options based on positive results from clinical trials. These new drugs, which are effective but expensive will increase the need for individualised prostate cancer care. One challenge, however, is how to match patient and drug in order to obtain the optimum treatment results at as low a cost as possible. Another challenge is how to decide when a drug has not worked at all, or is no longer effective.

Imaging biomarkers may be important tools in this context as prognostic and response indicators. Bone scan is the most common method for monitoring bone metastases in patients with advanced prostate cancer, e.g. in connection with treatment [8,9]. The interpretation of changes in the intensity and size of metastatic lesions on bone scans can be a difficult task and to some extent a subjective assessment causing variability between different readers. In an effort to make the interpretation more standardised, the Prostate Cancer Clinical Trials Working Group (PCWG2) has defined progression in bone as the presence of two or more new lesions on a bone scan compared with a prior scan [10]. If new lesions are present at the 12-week scan, the PCWG2 propose that a confirmatory scan is performed 6 or more weeks later and additional new lesions in this scan is considered evidence of progression. An alternative approach to quantifying the progression of metastatic disease is to calculate a bone scan index (BSI) reflecting the burden on the skeleton. The tumour burden is expressed as a percentage of the total skeletal mass. This method was recently evaluated in patients with prostate cancer receiving chemotherapy, and the results showed that on-treatment change in BSI was closely associated with overall survival [11]. The same study also showed that changes in PSA were not associated with survival, while adjusting for changes in BSI, indicating the value of BSI as a response indicator.

Visual image analysis for the detection of new lesions and the calculation of BSI is time consuming and subjective and involves inter-observer variability. We therefore recently presented an automated method for calculation of BSI [12]. The method was designed to analyse one scan at a time, and it was shown that the automated method provides important clinical information comparable to that of visual BSI scoring.

The aims of this study were twofold: firstly, to develop and evaluate an automated method for the detection of new lesions and changes in BSI in serial bone scans and secondly, to evaluate the prognostic value of the method in a group of patients on-treatment with docetaxel.

## Methods

### Patients

#### Evaluation group

The automated method for the detection of new lesions in serial bone scans was evaluated using a group consisting of all prostate cancer patients who, during the period January 2002 to December 2008, underwent two whole-body bone scan examinations using a dual-detector gamma camera at Sahlgrenska University Hospital, Gothenburg, Sweden. A total of 266 patients with a mean age of 76 years (range 47 to 97 years) were included. In patients with more than two scans, the last two scans were used.

#### Treatment group

The prognostic value of the automatically calculated number of new lesions and changes in BSI was evaluated retrospectively using the treatment group. This group consisted of all patients who during the period April 2005 to November 2008 received docetaxel (Taxotere; Sanofi-Aventis, Brussels, Belgium) for the treatment of metastatic hormone-refractory prostate cancer at Skåne University Hospital, Malmö, Sweden. A total of 31 patients underwent a baseline whole-body bone scan during the period 6 months before to 8 days after the start of the treatment, plus a follow-up bone scan during the period 3 to 12 months after the baseline bone scan. The patients had a mean age of 67 years (range 54 to 79 years) at the time of the baseline scan. The computerised medical records were updated until June 30, 2011. A total of 26 patients died during follow-up, with a median survival time from the follow-up bone scan of 17 months (interquartile range 9 to 21 months). The follow-up time for the five survivors ranged from 27 to 59 months after the follow-up scan.

The study was approved by the Regional Ethical Review Boards at Gothenburg and Lund Universities, Sweden.

### Bone scintigraphy

Bone scans were obtained approximately 3 hours after an intravenous injection of 600 MBq Technetium-99 m methylenediphosphonate (Tc-99 m MDP, Amersham, UK). Whole-body images, anterior and posterior views (scan speed 10 (evaluation group) or 15 (treatment group) cm/min, matrix 256 × 1024), were obtained using a gamma camera equipped with low-energy high-resolution parallel hole collimators (Maxxus; General Electric, Milwaukee, USA (evaluation group) or MultiSPECT2; Siemens Healthcare Diagnostics Inc. Deerfield, IL, USA (treatment group)). Energy discrimination was provided by a 15% window centred on the 140 keV of Tc-99 m.

### Automated method

**Figure 1:**
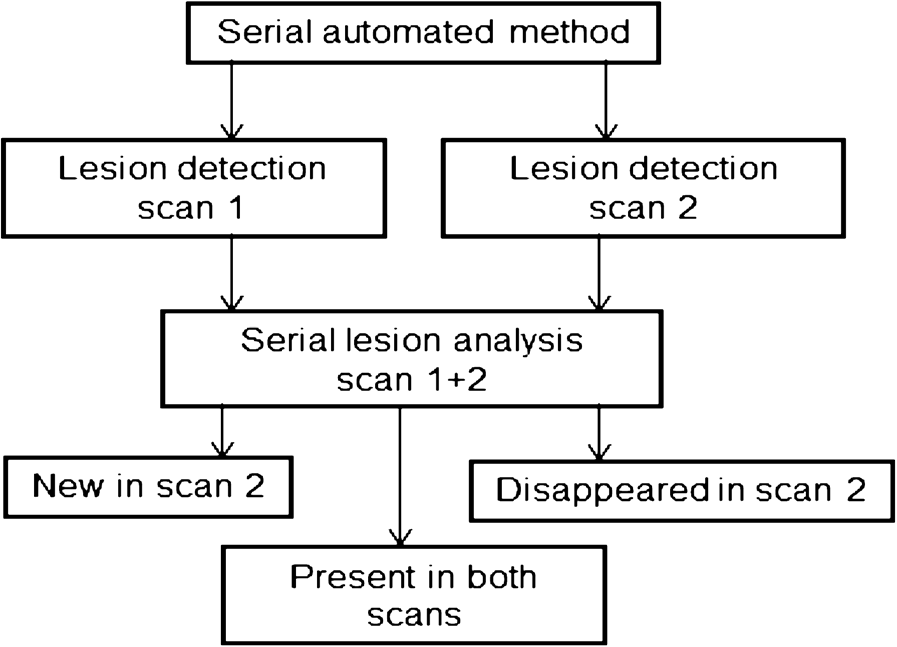
Automated detection of new lesions in serial whole-body bone scans.

We have previously presented a completely automated method that analyses single whole-body bone scans (the anterior and posterior images) one at a time [12]. BSI is calculated by first calculating the area of a hotspot classified as a metastatic lesion and then calculating the area of the corresponding skeletal region obtained from the segmentation of the skeleton (e.g. the skull or pelvis). Dividing the former by the latter and multiplying the result by a constant representing the weight fraction of the present skeletal region with respect to the weight of the total skeleton [13] gives an estimate of the volumetric fraction of the skeletal region occupied by the hotspot. This method, which is part of the EXINI bone software (EXINI Diagnostics AB, Lund, Sweden), has now been further developed so that a comparison of two whole-body bone scans from the same patient is carried out automatically (Figure 1).

**Figure 2:**
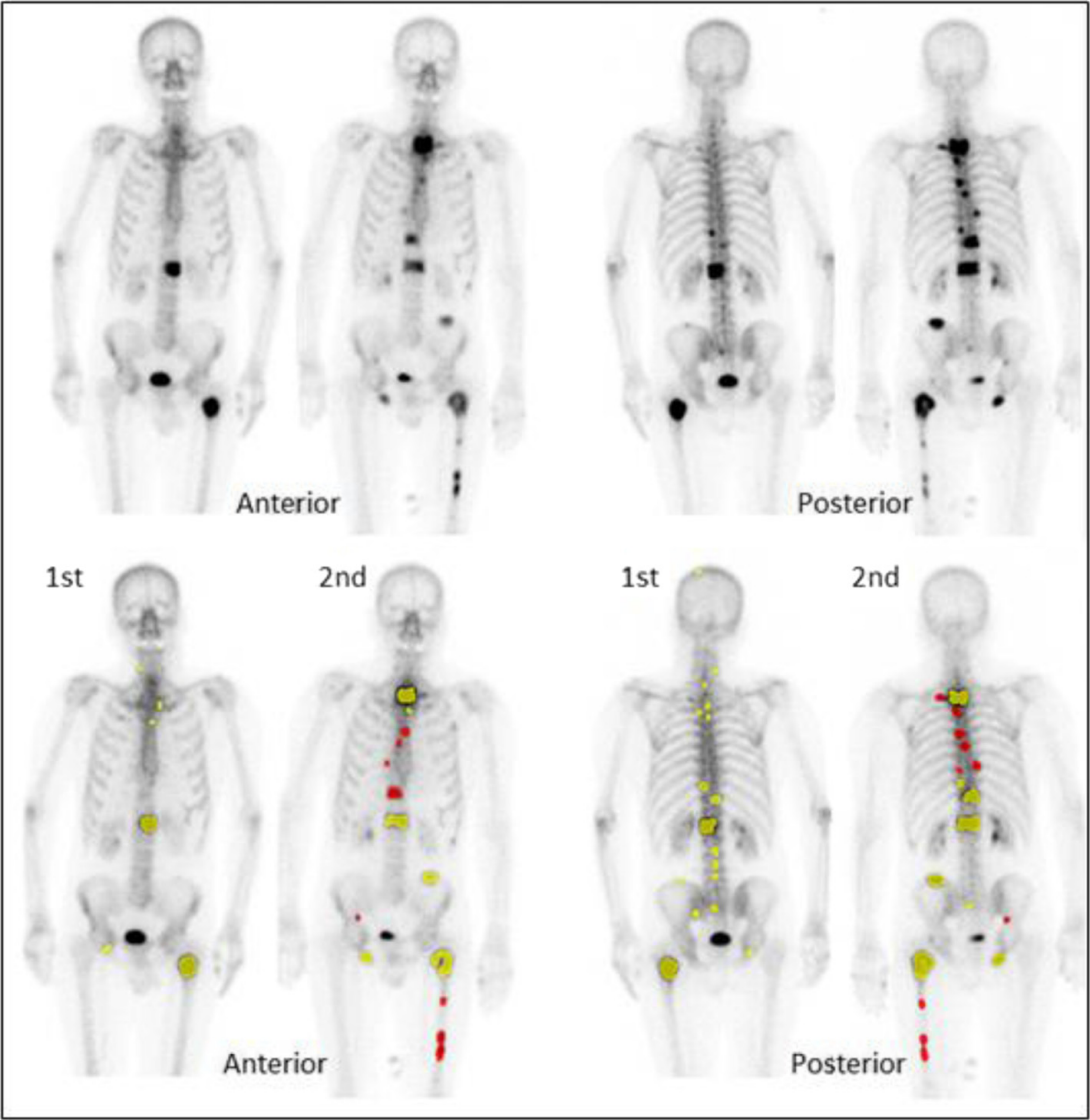
**Bone scans from a patient with progress of metastatic disease.** Anterior and posterior views from the first (1st) and second (2nd) scans without (above) and with (below) marks showing lesions detected by the automated method. Red marks indicate new lesions and yellow marks indicate old lesions.

A template normal scan is used to establish correspondences between time points. As part of the method presented in a previous study [12], the template scan is warped to fit each individual scan using a non-rigid registration algorithm. Separate warps are used for anterior and posterior images. We have developed a method for connecting a point in one scan to the corresponding point in other scans in the anatomical sense using these warps. The method uses the template scan as an intermediate step in this process, since scans at all time points share this coordinate frame. Using this technique, hotspots can be marked as connected across time points using a connected-component analysis based on measures of hotspot centroid proximity and hotspot overlap. Connecting hotspots which presumably originates from a single underlying skeletal region in this fashion greatly reduces the number of hotspots to review when examining multiple time points at once. Each group of connected hotspots is then assigned a single classification as metastasis/not metastasis by an automatic algorithm. New lesions that are not present in the previous scan are marked and counted. Figure 2 shows a patient with progress of the disease resulting in new lesions in the second scan.

### Visual evaluation

Visual analysis of all scans in the evaluation group was performed separately by three experienced bone scan readers. They independently classified all patients as either fulfilling the criterion of two or more new lesions, established by the PCWG2 [7], or not fulfilling it. The majority rule was applied in cases of disagreement, i.e. if at least two of the three readers found two or more new lesions in the follow-up scan the patient was defined as fulfilling the PCWG2 criterion. Clinical information, e.g. medical condition, localisation of bone pain and previous history of injury, was available to all the bone scan readers. In difficult cases, reports from other diagnostic examinations, e.g. follow-up scans using magnetic resonance imaging, X-rays or computed tomography, were considered in the re-evaluation.

### Statistical methods

Cox proportional hazards with 95% confidence intervals (CI) were used to investigate the association between percentage change in BSI (BSI_follow-up_/BSI_baseline_), number of new lesions and survival. Kaplan-Meier estimates of the survival function were used together with the log-rank test to indicate a significant difference between patients with an increase/decrease in BSI or two or more new lesions or less than two new lesions. A *p* value < 0.05 was considered significant.

## Results and discussion

### Results

At least two of the three readers found progression, i.e. at least two new lesions, in 120 patients and no progression in 135 patients. In 11 patients with very severe metastatic disease, the readers did not find it possible to decide about progression, and these cases were excluded. All three readers agreed in 87% (222/255) of the remaining cases, and in 13% of the cases, one found progression while the other two did not, or vice versa, i.e. several cases were difficult to classify as progression or no progression, even for experienced readers.

The automated method detected at least two new lesions in 112 of the 120 cases with progression, according to the experts, i.e. a sensitivity of 93%. In the remaining eight cases, the automated method detected one new lesion in each case. Among the 135 cases without progression according to the experts, the automated method showed a specificity of 87% (117/135). The corresponding negative and positive predictive values were 94% and 86%, respectively.

**Figure 3:**
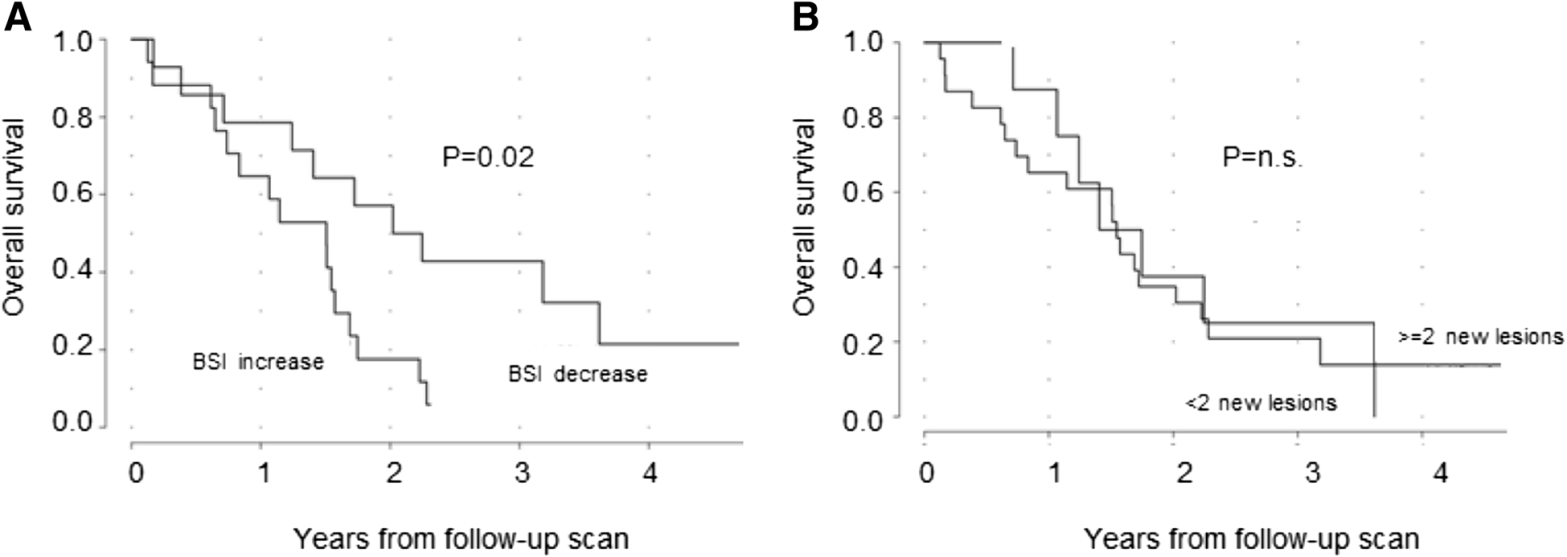
**Kaplan-Meier curve for overall survival in 31 men.** Stratified by **(A)** increase or decrease in BSI and **(B)** two or more new lesions or less than two new lesions in the follow-up scan.

The median change in BSI from the first to the second scan was an increase of 13% (range 59% decrease to 438% increase) in the treatment group. In a univariate Cox analysis, both the ‘percentage change in BSI’ (Hazard ratio 1.005; 95% CI, 1.001 to 1.008; *p* = 0.008) and the ‘number of new lesions’ (Hazard ratio 1.06; 95% CI, 1.02 to 1.09; *p* = 0.0004) were associated with survival. A total of 17 of the 31 patients in the treatment group showed an increase in BSI during treatment. Only three (18%) of these patients were alive 2 years after the second scan. Of the 14 patients with a decrease in BSI during treatment, eight (57%) were alive after 2 years. The Kaplan-Meier curves for patients with increase and decrease in BSI were significantly different (*p* = 0.02) (Figure 3A). Two new lesions were found in 23 patients, and eight (35%) of these patients were alive after two years. Three of the eight (38%) patients without two new lesions were alive after two years. The Kaplan-Meier curves for patients with and without at least two new lesions were not significantly different (Figure 3B).

### Discussion

The results of this study show that an automated method can be used to detect new lesions and changes in BSI in serial bone scans, and that these values contain prognostic information in a group of patients on-treatment with docetaxel. A sensitivity of 93% and a specificity of 87% for the automated method are high considering the inter-observer variability among human observers. Our gold standard was based on the classifications of three experienced observers, and they all agreed in 87% of the cases. Other studies have also shown substantial inter-observer variability. Sadik et al. studied bone scan classifications from 37 readers, and on average, found agreement between paired readers of 64% [14]. The lower value in that study can, at least partly, be explained by the fact that the classification was performed using a four-grade scale, and the fact that the readers came from 18 different hospitals. Ore et al., on the other hand, reported inter-observer agreement of 91% based on two observers in a smaller patient sample [15]. The difficulty of interpreting bone scan changes even for experienced readers is the incentive to develop an objective method in order to minimise disagreement among observers. Intra-observer variability was also found in our recent study in which one reader analysed the same bone scans twice on different occasions and calculated BSI visually [12].

In the treatment group, only 18% of the patients with an increase in BSI were alive after 2 years, while 57% of those with a decreasing BSI were alive after the same period. These results are in agreement with those of Dennis et al., who demonstrated the prognostic value of BSI as a response indicator in prostate cancer patients [11]. In this study, the PCWG2 criterion of two or more new lesions was not prognostic. The number of new lesions was, however, significantly associated with survival, indicating that a criterion other than two new lesions might be valuable. It might even be a combination of the number of new lesions and the percentage change in BSI that proves to contain the most prognostic information.

We used the subjective classifications by three experienced bone scan readers as the gold standard in the evaluation group. This is not an optimum gold standard, but no independent examinations of these patients were available that confirmed or excluded the presence of new lesions. We therefore added an evaluation group to assess the prognostic value of the automated measurements of new lesions and changes in the BSI.

The results of this study are based on a retrospectively selected group of clinical cases, and as a consequence, there is a lack of standardisation of the imaging times pre and post treatment. This lack of standardisation can be a confounding factor weakening the association between BSI and survival. In future prospective studies and retrospective studies based on cases from clinical trials, the time range between baseline scan and start of treatment as well as between baseline scan and follow-up scans should be more standardised, to strengthen the analysis.

A limitation of this study was that the treatment group was small and the analysis retrospective. The results are encouraging, but the clinical value of the automated quantitative analysis of serial bone scans needs to be confirmed in future studies. In a larger study, the imaging biomarkers, number of new lesions and percentage change in BSI could also be related to other biomarkers such as PSA. The PSA level and change during treatment are widely used, but it is well known that they are not reliable in castrate-resistant prostate cancer. Imaging biomarkers could, therefore, provide additional information in the management of prostate cancer patients.

Limitations of the bone scintigraphy method are also to be taken into consideration in a quantitative analysis. Flare response, especially early on in treatment, may result in misjudgement while measuring BSI. Lesions in a bone scan are non-specific, and they may be due to degenerative disease, fractures, etc. The automated software has proved to be as capable of interpreting bone scans and differentiating metastatic lesions from degenerative abnormalities as an experienced physician [16], but patient history is crucial to the detection of fractures after trauma.

## Conclusions

Our automated method for the detection of new lesions and changes in BSI in serial bone scans includes prognostic information in a group of patients on-treatment with docetaxel. These results indicate that an automated method could be used to minimise inter-observer variation in the analysis of bone scans, both in clinical routine and in clinical trials. BSI can be used as a response indicator in prostate cancer patients, but further studies are needed to confirm the results of this retrospective study.

## Competing interests

MO, KS and LE are shareholders in EXINI Diagnostics AB (Lund, Sweden) which provides EXINI bone, the automated software for analysis of bone scans. RK, PG, HL and ML indicated no potential conflicts of interest.

## Authors’ contributions

RK and LE participated in the design of the study and in the analysis and interpretation of data, and drafted the manuscript. HL, PG and ML participated in the design of the study and in the analysis and interpretation of data. KS and MO carried out the analysis of data. All authors read and approved the final manuscript.
